# Circulating microRNAs can predict chemotherapy-induced toxicities in patients being treated for primary breast cancer

**DOI:** 10.1007/s10549-023-07033-8

**Published:** 2023-08-04

**Authors:** Matthew G. Davey, Ray Abbas, Eoin P. Kerin, Maire Caitlin Casey, Andrew McGuire, Ronan M. Waldron, Helen M. Heneghan, John Newell, Ailbhe M. McDermott, Maccon M. Keane, Aoife J. Lowery, Nicola Miller, Michael J. Kerin

**Affiliations:** 1 Discipline of Surgery, Lambe Institute for Translational Research, University of Galway, Galway, H91 YR71 Ireland; 2 Royal College of Surgeons in Ireland, 123 St Stephens Green, Dublin, D02 YN77 Ireland; 3 School of Mathematics, Statistics and Applied Mathematics, University of Galway, Galway, H91 TK33 Ireland; 4 Department of Medical Oncology, Galway University Hospital, Galway, H71 YR71 Ireland; 5 Cancer Trials Ireland, Innovation House, Old Finglas Road, Dublin, D11 KXN4 Ireland

**Keywords:** Breast cancer, MiRNA, Chemotherapies, Precision oncology, Personalised medicine, Treatment toxicities

## Abstract

**Purpose:**

Prescribing NAC for breast cancer is a pragmatic treatment strategy for several reasons; however, certain patients suffer chemotherapy-induced toxicities. Unfortunately, identifying patients at risk of toxicity often proves challenging. MiRNAs are small non-coding RNA molecules which modulate genetic expression. The aim of this study was to determine whether circulating miRNAs are sensitive biomarkers that can identify the patients likely to suffer treatment-related toxicities to neoadjuvant chemotherapy (NAC) for primary breast cancer.

**Methods:**

This secondary exploratory from the prospective, multicentre translational research trial (CTRIAL ICORG10/11–NCT01722851) recruited 101 patients treated with NAC for breast cancer, from eight treatment sites across Ireland. A predetermined five miRNAs panel was quantified using RQ-PCR from patient bloods at diagnosis. MiRNA expression was correlated with chemotherapy-induced toxicities. Regression analyses was performed using SPSS v26.0.

**Results:**

One hundred and one patients with median age of 55 years were recruited (range: 25–76). The mean tumour size was 36 mm and 60.4% had nodal involvement (*n* = 61) Overall, 33.7% of patients developed peripheral neuropathies (*n* = 34), 28.7% developed neutropenia (*n* = 29), and 5.9% developed anaemia (*n* = 6). Reduced miR-195 predicted patients likely to develop neutropenia (*P* = 0.048), while increased miR-10b predicted those likely to develop anaemia (*P* = 0.049). Increased miR-145 predicted those experiencing nausea and vomiting (*P* = 0.019), while decreased miR-21 predicted the development of mucositis (*P* = 0.008).

**Conclusion:**

This is the first study which illustrates the value of measuring circulatory miRNA to predict patient-specific toxicities to NAC. These results support the ideology that circulatory miRNAs are biomarkers with utility in predicting chemotherapy toxicity as well as treatment response.

**Supplementary Information:**

The online version contains supplementary material available at 10.1007/s10549-023-07033-8.

## Introduction

Contemporary breast cancer management pragmatically uses neoadjuvant chemotherapy (NAC) as the standard of care in patients diagnosed with certain breast cancer molecular subtypes [1, 2]. While survival outcomes for patients with breast cancer are equivalent for those treated with adjuvant and NAC [3, 4], there are several advantages of prescribing NAC, including potential tumour downstaging, increasing patient eligibility for breast conservation surgery (BCS), and obtaining in vivo sensitivity of the tumour to treatment [5], which correlates with long-term survival [6]. Unfortunately, certain host and tumour factors make predicting treatment response challenging, and the risk of overtreatment is incessant [7]. Chemotherapeutic drugs are cytotoxic to human cells [8], and their administration adopts a untargeted approach, where therapies do not differentiate cancer cells from healthy host cells, leading to widespread toxicity [9]. Therefore, a proportion of patients receiving chemotherapies develop undesired treatment-related toxicities [10] and identifying such patients remains an ever-present challenge to the multidisciplinary team. Thus, the discovery of methods of identifying these patients is imperative to improve the care of our prospective patients being treated for cancer.

Micro ribonucleic acids (or miRNAs) are a contemporary class of small, non-coding ribonucleic acid (RNA) molecules which are estimated to be approximately 19–25 nucleotides in length [11]. It is now recognised that these biomolecules possess key modulatory roles in genetic expression by influencing the post-transcriptional degradation of messenger RNA [12, 13]. MiRNA expression profiles have been implicated as regulators of several cellular processes [14] and maintain their stability in several biological tissues, including tumour tissue, healthy host tissue, and human circulation [15]. Notwithstanding their ability to predict treatment response and estimate patient prognosis [16–18], there are emerging data suggesting miRNA may have roles in toxicities to cancer therapeutics, including chemoradiotherapies and immunomodulatory agents [19, 20]. Furthermore, the measurement of miRNAs may be performed relatively simply and inexpensively using real-time quantitative reverse transcriptase polymerase chain reaction (RQ-PCR) [21], which improves the clinical candidacy of these biomolecules as potential cancer and therapeutic biomarkers.

At present, there is a paucity of translational research studies which have successfully identified patients at an increased risk of chemotherapy-induced toxicities. The Cancer Trials Ireland—Irish Clinical Oncology Research Group 10/11 (CTRIAL ICORG 10/11) study was a prospective, multicentre clinical trial which recruited 120 patients indicated to undergo standard-of-care NAC from 8 treatment sites across the Republic of Ireland. In the primary analysis of the ICORG 10/11 trial, a predetermined miRNA panel was relatively quantified from bloods samples using RQ-PCR at several timepoints during NAC and correlated with treatment response to NAC [16, 22]. The current study is a secondary exploratory analysis of the ICORG 10/11 trial and, to the authors knowledge, is the first study to explore the clinical utility of miRNAs as circulatory biomarkers which may be useful in identifying patients who are at an increased risk of developing treatment-induced toxicities to NAC for primary breast cancer.

## Methods

### Study design

As outlined, the CTRIAL ICORG10/11 is a prospective, multicentre trial which recruited patients from 8 treatment sites in the Republic of Ireland (NCT01722851). Within ICORG 10/11, the primary objective measured was to decipher miRNA targets which could be used to predict treatment response to NAC. The current analysis is a secondary exploratory analysis which uses the data obtained in ICORG 10/11 and correlated these miRNA targets with chemotherapy-induced toxicities. This analysis was performed as per the Standard in Diagnostic Test Accuracy (or STARD) statement to determine the diagnostic test accuracy of miRNAs targets in predicting treatment-induced toxicities [23]. Following a formal power calculation performed by the School of Mathematics, Statistics, and Applied Mathematics at the University of Galway, it was established that 118 patients would require recruitment to accurately address the primary research measure (i.e.: response to NAC), leading to the initial recruitment and inclusion of 120 patients indicated to undergo standard-of-care NAC for primary breast cancer providing informed written consent. Thereafter, 19 of these patients subsequently did not have follow-up information available in relation to treatment-induced toxicities which led to their exclusion from this secondary analysis, leaving 101 patients to be included. Decisions regarding the chemotherapy regimens prescribed were decided based on the professional judgement of the multidisciplinary team in each local tertiary referral centre for breast cancer management. Treatment decisions were made by the breast cancer multidisciplinary team in accordance with internationally accepted standards and guidelines (i.e.: those from the European Society for Medical Oncology and National Comprehensive Cancer Network) [24, 25]. The most common chemotherapy-induced toxicities reported in the seminal TAILORx and RxPONDER prospective, randomised clinical trials were used for comparison in this study [26, 27].

### Research ethics

Ethical approval was prospectively obtained from the Galway University Hospitals (C.A.151-February 2008) and University of Galway Clinical Research Institutional boards (C.A.1012-January 2014). In addition, local hospital ethical approval was also obtained from each of the participating centres responsible for patient recruitment.

### Participant inclusion and exclusion criteria

Consecutive female patients aged 18 years or older diagnosed and treated for primary breast cancer who were indicated to undergo NAC in accordance with best practice guidelines were considered for inclusion in the current study. All patients had to be capable of providing informed written consent and had to have follow-up data pertaining to treatment-induced toxicities available. Patients were excluded from this study if failed to meet this outlined inclusion criteria.

### Tumour profiling and staging

Tumour specimens underwent classification into breast cancer molecular subtypes using the 11th St. Gallen Expert Consensus panel [28], based on the work of Perou et al. [29]. Specimens were analysed as per the 2010 American Society of Clinical Oncology/College of American Pathologists (ASCO/CAP) histopathological consensus guidelines for oestrogen (ER) and progesterone (PgR) receptor status using immunohistochemistry [30, 31]. Human epidermal growth factor receptor-2 (HER2) receptor status was delineated using Herceptest™ (DAKO Agilent pathology solutions, Santa Clara, CA, USA), where 3 + was considered to be HER2-positive, with 2 + inconclusive results confirmed using fluorescent in situ hybridisation testing [32, 33]. Appraisal of Ki-67 was performed using MIB1 antibody testing [34, 35].

In brief, luminal cancers (LBC) possessed ER and PgR positivity with HER2 negativity (ER + /PgR + /HER2 −), luminal B-HER2-positive cancers (LBBC-HER2 +) possessed ER + and HER2 positivity with variable PgR expression (ER + /HER2 +), HER2 cancers (HER2 +) possessed ER and PgR negativity with HER2 positivity (ER −/PgR −/HER2 +), and triple negative cancer (TNBC) possessed ER, PgR , and HER negative disease (ER −/PgR −/HER2 −). Tumour staging was performed as per the American Joint Committee on Cancer (AJCC), version 8 guidelines [36].

### Venous blood sampling

Venous blood samples from the 101 included patients were collected over a 3-year period (May 2011–April 2014). Whole blood liquid biopsies were collected at five independent timepoints. For this analysis, samples obtained at the time of breast cancer diagnosis (prior to treatment with NAC) were used. Venous blood samples were collected in ethylenediaminetetraacetic acid (EDTA) tubes and stored at the Organisation of European Cancer Institutes (OECI) approved Comprehensive Cancer Centre Biobank at the Department of Surgery at the University of Galway (UG).

### MiRNA expression panel

Based on their previous reported and perceived relevance to breast cancer (i.e.: ontogenetic or tumour suppressor properties), a panel of five miRNAs was selected for evaluation (Let-7a, miR-21, miR-145, miR-155, and miR-195) [37–39]. The relevance and rationale for selecting these miRNA targets are illustrated in Table 1**.** It is important to note that it was initially perceived that miRNAs had only one primary function [37]; however, it is now understood that these biomarkers are multifunctional and may have additional roles in human biology following the administration of cytotoxic chemotherapy that have not previously described (e.g.: immunomodulatory function in bone marrow suppression or inflammation). Therefore, the hypothesis suggesting that these miRNA targets may have credibility in predicting treatment-induced toxicities to NAC is plausible. Two miRNAs (miR-16 and miR-425) were selected and used as validated endogenous controls due to their stability in the circulation of breast cancer patients. These targets were then used to calibrate samples between patients [18, 40]. The relevance of the miRNA selected for inclusion in the panel for this study is outlined in Table 1.

**Table 1:** Predetermined miRNA panel consisting of the 7 miRNAs (5 targets and 2 endogenous controls) included in this study and their rationale for selection

MicroRNA	MiRNA function
Let-7a	Increased expression in breast cancer patients compared to controls. Reduced expression in patient’s post-resection compared to with active disease (Heneghan et al. [37])
miR-21	Oncogenic miRNA (Heneghan et al. [38])
miR-145	Increased expression in breast cancer compared to controls (Heneghan et al. [37])
miR-155	Differentiated expression in breast cancer compared to controls (Heneghan et al. [37])
miR-195	Increased expression in breast cancer compared to controls (Heneghan et al. [37])
miR-16	Endogenous control in human circulation (McDermott et al. [40])
miR-425	Endogenous control in human circulation (McDermott et al. [40])

### RNA isolation and storage

Total RNA was extracted from whole blood (1 mL) using Trizol (as per the manufacturer’s instructions). RNA concentrations were determined using spectrophotometry (NanoDrop ND-1000 Technologies Inc., Wilmington, DE, USA) [37, 38]. RNA was then transferred to storage tubes and labelled and stored at − 70 °C in the Cancer Biobank at the University of Galway.

### Analysis of miRNA expression levels

For each blood sample, miRNAs were relative quantified using polymerase chain reaction (RQ-PCR). TaqMan assays were used, in accordance with the manufacturer’s instructions, for the (RQ-PCR) of the indicated target miRNA (miRNA: Taqman assay ID- miR-195: 000494; miR-155: 002623; miR-145: 002278; miR-21: 000397; Let-7a: 000377; miR-10b: 002218) and the endogenous control (miR-16: 000391; miR-425: 001104), as previously described (TaqMan Fast Universal Master Mix (2X), No AmpErase UNG: Applied biosystems, Foster City, CA, USA, cat:4367846) [11, 40]. Assays were performed using an AB7900HT (Applied Biosystems), using standard conditions as per manufacturer’s instructions. Reactions were commenced with a 10-min incubation at 95 °C before being followed by 40 cycles at 95 °C for 15 s and 60 °C for 60 s. MiR-26b was utilised as an inter-assay control which was derived from a breast cancer cell line. These were included on each plate for calibration. To ensure reproducibility to account for outliers, reactions were performed in triplicate (with each individual assay performed using technical triplicates). The threshold standard deviation (SD) for intra-assay and inter-assay replicates was 0.3. The percentage of PCR amplification efficiencies (*E*) for each assay were calculated using the slope of the semi-log regression plot of cycle threshold vs. log input of cDNA (tenfold dilution series of five points), with the following equation, and a threshold of 10% above or below 100% efficiency was applied: $E\, = \,\left( {{1}0^{{ - {1}/{\text{slope}}}} \, - \,\,{1}} \right)\, \times \,{1}00$. Moreover, miRNA expression levels were calibrated and normalised using endogenous controls. Thereafter, miRNA expression levels were calculated using QbasePlus© software (Biogazelle, Gent, Belgium) using the geNorm method to ensure the results were calibrated and normalised before being relatively quantified compared to the endogenous controls (miR-16 and miR-425). MiRNA analysis was performed blinded to clinicopathological data.

### Definitions of treatment response and toxicity

- Treatment response to NAC was measured using the Miller-Payne classification system, as outlined initially by Ogston et al. [41].
- Anaemia; National Cancer Institute Anaemia Scale of grade 2 or worse (i.e.: red blood cell concentrations of 10 g/mL or less) due to chemotherapy-induced bone marrow suppression [42].
- Neutropenia; the first laboratory evidence of reduced neutrophil count (neutrophil counts less than 2.5 g/mL) in a patient receiving chemotherapy, as per the local hospital guidelines.
- Neutropenic sepsis; developing pyrexia (temperature 38.0 °C) combined with a neutrophil count of less than 2.5 g/mL while receiving NAC, as per the National Institute for HealthCare Excellence [43].
- Peripheral neuropathy; chemotherapy-induced neurological symptoms including paraesthesia, paralysis, or neuropathic pain [44].

### Test methods and statistical analysis

Regression analyses were performed to correlate miRNA expression with toxicities to NAC, with associated 95% confidence intervals (95%CIs) reported in accordance with the ‘statistical analysis and methods in the published literature’ (or SAMPL guidelines) as previously described by Lang et al.’ [45]. The index test for this analysis was miRNA expression profiles (expressed as continuous data) which were compared to the clinical outcome measure of whether patients developed chemotherapy-induced toxicities (expressed as binary data) [23]. All analyses were two-tailed and statistical significance was defined as *P* < 0.050. Data were analysed using statistical package for social sciences (SPSS) version 26 (International Business Machines Corporation, Armonk, New York).

## Results

### Clinicopathological data

In this study, 101 patients were prospectively recruited. The median age at diagnosis was 55.0 years (range: 25.0–76.0) and the mean tumour size was 36.0 mm (range: 10.0–100.0 mm). The majority had nodal involvement (60.4%, *n* = 61) and 99.0% patients had grade 2/3 disease (*n* = 100), Overall, 46.5% had luminal (LBC, *n* = 47), 17.8% had luminal B-HER2 + (LBBC-HER2 + , *n* = 18), 15.8% (HER2 + , *n* = 16), and 18.8% had triple negative (TNBC, *n* = 19).

### Neoadjuvant chemotherapy

Overall, 30.7% of patients achieved a pCR (31/101). All 101 patients completed their anticipated NAC regimens (100.0%). Over 50% of patients received doxorubicin and cyclophosphamide followed by paclitaxel (AC-T) (55.5%, *n* = 56) (Supplementary Material S1). Basic demographic, clinicopathological, and treatment data for the 101 patients included are outlined in Table 2. Exact time interval between index test (i.e.: venous sampling for miRNA expression) and clinical interventions (i.e.: NAC administration) was typically less than 4 weeks.

**Table 2:** Basic demographic, clinicopathological, and treatment data for all 101 included patients

Parameter	Variable	Total
Total number	–	101 (100.0%)
Age (Years)	Median (Range)	55 (25–76)
Tumour size (mm)	Median (Range)	36 (10–100)
Nodal involvement	Negative	40 (39.6%)
	Positive	61 (60.4%)
Tumour grade	Grade 1	1 (1.0%)
	Grade 2	52 (51.5%)
	Grade 3	48 (47.5%)
Oestrogen receptor	Positive	63 (62.4%)
	Negative	38 (37.6%)
Progesterone receptor	Positive	54 (53.5%)
	Negative	37 (46.5%)
HER2 receptor	Positive	34 (33.7%)
	Negative	67 (66.3%)
Molecular subtype	LBC	47 (46.5%)
	LBBC-HER2	18 (17.8%)
	HER2 +	16 (15.8%)
	TNBC	19 (18.8%)
Response to NAC	pCR	31 (30.7%)
	Residual disease	70 (69.3%)
NAC regimen	AC-T	56 (55.5%)
	TC-H	19 (18.8%)
	TC-HL	6 (5.9%)
	Other	20 (19.8%)
Surgery	BCS	54 (53.5%)
	Mastectomy	47 (46.5%)
Axillary surgery	SLNB only	28 (27.7%)
	ALNB	73 (72.3%)

*HER2* human epidermal growth factor receptor-2, *LBC* luminal breast cancer, *LBBC-HER2* luminal B breast cancer, *HER2 + * human epidermal growth factor receptor-2 positive molecular subtype, *TNBC* triple negative breast cancer, *NAC* neoadjuvant chemotherapy, *pCR* pathological complete response, *AC-T* Doxorubicin and cyclophosphamide followed by paclitaxel, *TC-H(L)* docetaxel, carboplatin followed by trastuzumab (and lapatinib), *BCS* breast conservation surgery, *SLNB* sentinel lymph node biopsy, *ALND* axillary lymph node dissection

### Chemotherapy-induced toxicities

During treatment with NAC, 33.7% of patients developed symptoms of peripheral neuropathies (*n* = 34), 26.7% developed nausea and vomiting (*n* = 27), 5.9% developed myalgia (*n* = 6), and 11.9% developed mucositis (*n* = 12). With respect to bone marrow suppression, 28.7% developed neutropenia (*n* = 29), 59.4% developed lymphopenia (*n* = 60), and 5.9% developed anaemia (*n* = 6). Importantly, 2.0% of patients developed neutropenic sepsis (*n* = 2).

### MicroRNA predicting chemotherapy-induced toxicities

Reduced expression of miR-195 predicted patients likely to develop neutropenia during NAC (OR 0.344, 95%CI 0.111–0.990, *P* = 0.048), while increased expression of miR-10b predicted patients likely to develop anaemia (OR 0.038, 95%CI 0.001–0.910, *P* = 0.049). Moreover, increased expression of miR-145 predicted patients likely to develop nausea and vomiting during NAC (OR 3.819, 95%CI 1.252–11.652, *P* = 0.019), while decreased expression of miR-21 predicted patients likely to develop mucositis (OR 0.251, 95%CI 0.090–0.699, *P* = 0.008). Table 3 illustrates miRNA and their correlation with toxicities to NAC.

**Table 3:** Logistic regression analyses to determine the correlation of MiRNA expression profiles and developing toxicities to neoadjuvant chemotherapy

	Neutropenia	Lymphopenia	Anaemia	Neutropenic Sepsis	Peripheral Neuropathy	Nausea and Vomiting	Myalgia	Mucositis
Let-7a	0.846 (0.386–1.852), 0.675	0.557 (0.133–2.335), 0.424	1.188 (0.324–4.353), 0.795	3.129 (0.315– 31.084), 0.330	1.638 (0.823–3.258), 0.160	0.724 (0.377–1.391), 0.333	0.594 (0.199–1.776), 0.351	0.399 (0.169–0.944), 0.036
miR-10b	0.363 (0.085–1.547), 0.171	0.301 (0.033–2.761), 0.289	0.038 (0.001–0.998), 0.021*	103.233 (0.584–18,242.287), 0.079	0.579 (0.155–2.167), 0.417	0.630 (0.147–2.706), 0.534	0.601 (0.120–1.901) 0.181	2.844 (0.293–27.586), 0.367
miR-21	1.102 (0.516–2.353), 0.802	0.282 (0.046–1.748), 0.174	1.891 (0.404–8.843), 0.418	13.786 (0.390–487.344), 0.149	0.864 (0.427–1.749), 0.684	0.621 (0.296–1.300), 0.206	0.515 (0.125–2.121), 0.358	0.251 (0.090–0.699), 0.008*
miR-145	0.871 (0.379–2.000), 0.744	0.535 (0.103–2.775), 0.456	2.181 (0.368–12.945), 0.391	5.620 (0.155–203.582), 0.346	1.340 (0.604–2.972), 0.472	3.819 (1.252–11.652), 0.019*	0.400 (0.108–1.485), 0.171	0.725 (0.264–1.987), 0.532
miR-155	1.651 (0.801–3.402), 0.174	1.364 (0.642–2.901), 0.420	1.071 (0.397–2.888), 0.893	3.256 (0.476–22.271), 0.229	1.483 (0.775–2.839), 0.234	1.450 (0.731–2.877), 0.288	0.929 (0.316–2.731), 0.893	0.684 (0.359–1.302), 0.247
miR-195	0.344 (0.111–0.990), 0.048*	0.832 (0.174–3.976), 0.817	0.306 (0.091–1.029), 0.056	5.771 (0.109–305.796), 0.387	2.043 (0.721–5.794), 0.179	0.805 (0.294–2.207), 0.674	0.234 (0.040–1.362), 0.106	0.649 (0.179–2.359), 0.512

Results are expressed as odds ratios with 95% confidence intervals in brackets, followed by *P*-values

*Denotes statistical significance

## Discussion

Previous studies [12], including the primary analysis of the ICORG 10/11 clinical trial [16, 22], have successfully proved that circulating miRNAs have clinical utility in predicting tumour sensitivity to neoadjuvant therapies in patients with breast cancer. At the time of writing, this is the first study to our knowledge which successfully assessed the viability of miRNAs in predicting patients at risk of undesirable treatment-induced toxicities to NAC. The ideology of precision oncology focuses on maximising toxicity to the tumour, while minimising harm to the patient, which sets the foundations for the current analysis. Notwithstanding this, conventional cancer management seems to focus largely on predicting tumour responses to NAC (which has previously been demonstrated the carry a robust survival advantage for the majority) [6, 46, 47], while failing to identify factors indicative chemotherapy-induced toxicity. This study attempts to readjust the focus of translational research studies towards the prediction of patient-specific responses to NAC and to recentre the host (or patient) at the core of treatment paradigm. Therefore, the dogma presented by precision oncology is well captured within the current study, through the provision of novel results illustrating miRNAs as circulatory biomarkers capable of predicting treatment toxicities to NAC. Thus, this study is the first to describe such findings in the oncological literature and will hopefully provide direction for the next generation of translational research studies.

Chemotherapy-induced neutropenia is a major dose-limiting toxicity in clinical oncology and is renowned for placing significant burden upon healthcare economies globally [48]. Identifying patients at risk of this toxicity remains a clinical conundrum. In this study, patients with reduced expression of circulating miR-195 were significantly more likely to develop treatment-induced neutropenia than their counterparts. Aberrant expression of miR-195 has traditionally been recognised as a oncogenic biomarker in breast cancer [38, 49] and there are now emerging data indicating that miRNAs play a regulatory role in circulatory neutrophil proliferation [50]. This is the first report associating miR-195 with myelosuppressive neutropenia, which coincides with the previous data demonstrating miR-195 to be a stress-inducible target with potential immunomodulatory function [51–53]. In humans, the miR-195 gene encodes from the reverse strand of the mRNA gene (AK098506), which is responsible for encoding the LOC284112 hypothetical protein, which may have a protective role against bone marrow suppression [54, 55]. While this finding correlating miR-195 expression with treatment-induced neutropenia is one of the significant novelties, we acknowledge that these preliminary results will require further robust interrogation and validation prior to having clinical impact upon therapeutic decision-making in the treatment of cancer patients.

Chemotherapy-induced anaemia has a prevalence of greater than 40% in patients being treated with chemotherapy for breast cancer [56], thus ratifying the necessity for the early detection in this patient cohort. In this study, increased miR-10b was observed in patients who were at an increased risk of developing anaemia during NAC. MiR-10b has been identified to be an oncomir with perceived function in the metastatic cascade in breast cancer [57]; however, previous reports have illustrated the influence of miR-10b on toxicities to chemoradiotherapy in patients with primary glioblastoma, with miR-10b/miR-21 expression correlating with the degree of treatment toxicity [58], an analysis not performed in the current study. Moreover, miR-10b dysregulation has been previously implicated as causative in myelosuppressive disorders [59], making it plausible that increased expression of miR-10b may negatively influence circulatory haemoglobin levels in the post-chemotherapy effect, as illustrated in the current analysis of patients with breast cancer.

In this analysis, increased miR-145 predicted patients likely to develop gastrointestinal dysfunction (i.e.: nausea and vomiting) to NAC, while decreased expression of miR-21 predicted those likely to suffer from the development of mucositis. Inflammation of the gastrointestinal and oral mucosa is a significant negative implication for patients in receipt of chemotherapeutic agents [60, 61]. These provisional results yield promise for the early identification of such patients, which may facilitate early pharmacological prophylaxis. Notwithstanding the inevitability that further scientific assessment is necessary before validating these promising results, these findings may also be used to broaden the horizon to include NAC-induced toxicities within the primary or secondary outcome measures assessed within the next generation of biomarker discovery trials. This is particularly pertinent as current trials often fixate solely upon treatment response as sole analytical endpoints, thus failing to consider the patient-related issues associated with NAC.

This study suffers from several limitations: Firstly, the miRNAs evaluated were included in a predetermined miRNA panel which was decided upon at the time of study design over a decade ago, based on their perceived relevance to breast oncology at that time [37–39]. In the time that has elapsed, newer, potentially more relevant miRNAs may have been subsequently discovered. Secondly, this study measures miRNAs from circulation only, failing to evaluate tumour miRNAs from pre-treatment biopsies, meaning tissue yielding crucial genetic information was missed during the tissue acquisition phase of ICORG 10/11. Thirdly, breast cancer is a heterogeneous disease with several biomolecular subtypes with varying treatment algorithms [1], some of which will have evolved since patient recruitment commenced to this study. These factors limit the robustness of these results in present-day contemporary practice. Furthermore, the primary outcome measure of ICORG 10/11 was not powered to provide definitive results among subgroup analyses limiting the robustness of results. Accordingly, the authors have pragmatically not performed subgroup analyses in this secondary exploratory analysis to prevent potential dilution of the robustness of results yielded. Finally, we must highlight these are preliminary results which will inevitably require validation before translation into the contemporary management of breast cancer.

In conclusion, this secondary exploratory analysis is the first from a prospective, multicentre, neoadjuvant translational research trial which successfully assesses the value of measuring circulatory miRNAs to predict patient-specific toxicities to NAC. These results support the persistent ideology that miRNAs may be useful biomarkers with utility in personalising treatment algorithms in accordance with the needs of each patient, while also focusing on patient-specific responses, as well as tumour responses. Thus, this secondary exploratory analysis sets the foundations for the next generation of miRNA clinical trials should be designed to evaluate the use of circulatory miRNAs to predict the treatment-induced toxicities to NAC to ensure patient monitoring is individualised for prospective patients.

## Supplementary Information

Below is the link to the electronic supplementary material.

Supplementary file1 (DOCX 13 kb)

## Data Availability

Data will be made available upon reasonable request from the senior author, MJK.
