# Supplementary material for: Circulating microRNAs can predict chemotherapy-induced toxicities in patients being treated for primary breast cancer
Source: Breast Cancer Res Treat. 2023 Aug 4;202(1):73–81. doi: 10.1007/s10549-023-07033-8 (PMC10504160; doi:10.1007/s10549-023-07033-8)
Supplement: Supplementary file 1 — Supplementary file1 (DOCX 13 kb) [file 10549_2023_7033_MOESM1_ESM.docx]

**Circulating MicroRNAs Can Predict Chemotherapy-Induced Toxicities in Patients Being Treated for Primary Breast Cancer**

Matthew G. Davey^1^, Ray Abbas^1^, Eoin P. Kerin^1^, Maire Caitlin Casey^1^, Andrew McGuire^1^, Ronan M. Waldron^1^, Helen M. Heneghan^1^, John Newell^2^, Ailbhe M. McDermott^1^, Maccon M. Keane^3^, Aoife J. Lowery^1^, Nicola Miller^1^, Michael J. Kerin*^1,4^

^1^Discipline of Surgery, Lambe Institute for Translational Research, University of

Galway, Galway H91 YR71, Ireland

^2^School of Mathematics, Statistics and Applied Mathematics, University of Galway, Galway H91 TK33, Ireland

^3^Department of Medical Oncology, Galway University Hospital, Galway H71 YR71, Ireland

^4^Cancer Trials Ireland, Innovation House, Old Finglas Road, Dublin D11 KXN4, Ireland

*European Surgical Association Member Sponsor

**SUPPLEMENTARY MATERIAL**

**Supplementary Material S1.**

Detailed outline of the chemotherapy regimens prescribed in the current study.

| **Chemotherapy regimen** | **Number (%)** |
| --- | --- |
| Doxorubicin and cyclophosphamide followed by paclitaxel (AC-T) | 56 (55.5%) |
| Docetaxel, Carboplatin and Trastuzumab (TCH) | 19 (18.8%) |
| Docetaxel, Carboplatin and Trastuzumab, Lapatinib (TCHL) | 7 (6.9%) |
| Other combinations | 19 (18.8%) |
